# Isolated rural general practice as the focus for teaching core clinical rotations to pre-registration medical students

**DOI:** 10.1186/1472-6920-5-22

**Published:** 2005-06-27

**Authors:** Stephen A Margolis, Llewellyn M Davies, Valmae Ypinazar

**Affiliations:** 1 School of Medicine, University of Queensland, Brisbane, Australia

## Abstract

**Background:**

Earlier studies have successfully demonstrated that medical students can achieve success in core clinical rotations with long term attachments in small groups to rural general / family practices.

**Methods:**

In this study, three students from a class of 226 volunteered for this 1-year pilot program, conducted by the University of Queensland in 2004, for medical students in the 3rd year of a 4-year graduate entry medical course. Each student was based with a private solo general practitioner in a different rural town between 170 and 270 km from the nearest teaching hospital. Each was in a relatively isolated rural setting, rated 5 or 6 on the RRMA scale (Rural, Remote, Metropolitan Classification: capital city = 1, other metropolitan = 2, large regional city = 3, most remote community = 7). The rural towns had populations respectively of 500, 2000 and 10,000. One practice also had a General Practice registrar. Only one of the locations had doctors in the same town but outside the teaching practice, while all had other doctors within the same area. All 3 supervisors had hospital admitting rights to a hospital within their town. The core clinical rotations of medicine, surgery, mental health, general practice and rural health were primarily conducted within these rural communities, with the student based in their own consulting room at the general practitioner (GP) supervisor's surgery. The primary teacher was the GP supervisor, with additional learning opportunities provided by visiting specialists, teleconferences and university websites. At times, especially during medicine and surgery terms, each student would return to the teaching hospital for additional learning opportunities.

**Results:**

All students successfully completed the year. There were no statistical differences in marks at summative assessment in each of the five core rotations between the students in this pilot and their peers at the metropolitan or rural hospital based clinical schools.

**Conclusion:**

The results suggest that isolated rural general practice could provide a more substantial role in medical student education.

## Background

The last ten years have seen a global change in the methods used for the education and training of medical students [1-3]. In part, this has been due to the concurrent change in the operations of the tertiary level hospital system, traditionally the key learning site for the medical student. Hospitals are increasingly becoming short stay institutions with a focus on same day treatments and early discharge, with only the more seriously sick patient staying for a longer time [3-5]. This change of priorities has meant that students in tertiary teaching hospitals no longer have access to a full range of educational experiences as many of the less severe illnesses and other chronic illness move to the primary health care system [5-7].

One result of these changes has been a large scale move towards increasing community based medical education within the medical schools' curriculum, with students attached to a community based general / family practice [3,6-8]. Although these are usually relatively short placements of approximately 8 to 12 weeks [4,9], some medical schools have been providing a full year's study in the community [2,5,10,11].

An additional benefit of placement in the community is the increased exposure to general / family practice. When the community is in a rural area, this has a positive influence on choice of rural practice as a career choice, an area of significant shortage in many countries [9,12-14]. There are also positive benefits to established rural practitioners, with enhanced job satisfaction and consequently a positive impact on rural retention [7].

Initial concerns of both students and teachers experiencing long term placements in the community focused on the potential disadvantage to students because of the uncertainty of whether they would see a broad spectrum of clinical conditions through the community patient base to achieve their learning objectives [8,10]. However, there are also advantages such as experiencing longitudinal care [5,11], the opportunity to see early presentation of conditions, preventative medicine and an understanding of the psychosocial aspects of primary health care [5]. Community-based students also have the advantage of small group learning as they are generally working in groups of 2 to 4 students [2,11]. This obviates concerns regrading peer-to-peer interaction [15]. Early concerns regarding access to resources have by and large been resolved with appropriate Internet technology and equipment being made available within the community teaching sites [16,17].

Recent studies examining assessment results indicated that students in longitudinal community placement performed as well, if not better than their hospital-based counterparts in acquisition of clinical skills, including those associated with specialist disciplines [2,11]. Alderson and Oswald reported that students recorded a large number of patient contacts with varied clinical conditions representing problems in all major specialty areas [8].

Hence, earlier studies have successfully demonstrated that medical students can achieve success in core clinical rotations with long term attachments in small groups to rural general / family practices. The aim of this pilot study was to assess the feasibility of successfully completing the five third year core clinical rotations while based alone without their peers in a relatively isolated rural community general / family practice, with success measured though academic achievement.

## Methods

### Setting

The University of Queensland medical course is a four-year graduate entry program culminating in a Bachelor of Medicine and Bachelor of Surgery. The overall course plan is detailed in table 1. Students spend the first two years at the university campus in the capital city and then elect to attend one of three clinical divisions. Two are located in the metropolitan area, and one, the Rural Clinical Division, is based over two regions in rural Queensland. Twenty five percent of the School's students complete at least one of the last two years of the MBBS course in the Rural Clinical Division, with almost all of these students being volunteers. All terms for a particular year are completed within the chosen Clinical Division. All of the School's students are placed in a rural area to complete a third year term in rural health.

**Table 1:** Course structure

Year	Location	Subjects
1	University: main campus	Introduction to Medical Practice
2	University: main campus	Foundations of Medical Practice
3	Rural or Metropolitan Clinical School*	Medicine
		Surgery
		Mental Health
		General Practice
		Rural Health *
4	centerRural or Metropolitan Clinical School	Paediatrics
		OBGYN
		Surgical specialties
		Medical specialties

* Except Rural Health which all students do in rural areas

Problem Based Learning (PBL) is a key component of the program at all levels. Using a series of carefully planned patient-centered exercises, the students work cooperatively with their tutors to examine core issues of health and disease. In years three and four, students examine a new PBL case each week. The remainder of their clinical time is focused on supervised learning from their patients, supplemented by clinical teaching, tutorials, case demonstrations and lectures. Adult learning processes are encouraged with students' teaching and learning driven by their own learning objectives and goals. Key features of the program include life long learning skills including an ability to search and critically analyze information.

All students are learning under the same curriculum and participate in the same assessment process, regardless of the geographical location of their clinical experience. A summative mark is generated for each core clinical rotation in third year from an end-of-term test with or without an in-course assessment. From the rotation marks, an average score for the year is produced.

### The pilot program

The concept was initially developed at a stakeholder meeting of clinical and academic staff of the Central Queensland Rural Clinical Division held in early 2003. Several rural general practitioners (GP) who were clinical teaching staff at this Division expressed interest in developing and participating in the program. By late 2003, three rural GPs had agreed to participate.

In August / September 2003, the second year student body was approached at a series of public meetings and offered participation in the new program. Those students who were interested were asked to provide a confidential written expression of interest, explaining their motivation to participate, although there were no formal criteria to evaluate this process. The three students who expressed interest attended a weekend orientation program visiting all three prospective locations, meeting with their prospective GP supervisors, as well as the rural teaching hospital. All three subsequently agreed to volunteer for the program and self selected their site for attachment for 2004.

Program coordination was by a dedicated team based at the Division's rural teaching hospital. The attached academic staff also provided support both by telephone and regular visits.

Each student was attached to a different private solo rural general practitioner / family doctor for one academic year. Each student's home base was their own consulting room located within the community based General Practice. The practices are located in relatively isolated and geographically separated communities, all a considerable distance from each other. They are between 170 and 270 km from the Rural Clinical Division teaching hospital. The demographics of these locations are detailed in table 2. The locations are rated 5 or 6 on the RRMA scale (Rural, Remote, Metropolitan Classification: capital city = 1, other metropolitan = 2, large regional city = 3, most remote community = 7 [18]). The towns had respective populations of 500, 2000 and 10,000. One practice had a GP registrar and in one other location there were doctors outside the teaching practice but in the same town. All 3 supervisors had hospital admitting rights to a community hospital within their town.

**Table 2:** Demographics of pilot study locations

Location	RRMA [18]	ARIA [18]	Distance to (km):			Public Hospital Facilities (2002 -3) [24]			
			Division Teaching Hospital	Metropolitan Capital	Nearest Pilot Study Location	Available beds	Medical staff [FTE]	Visiting medical staff [FTE]	Admitted patients (in 2002)

A	5	4.03	172	640	78	10	1	0	463
B	5	4.79	210	564	78	10	1	0	291
C	6	5.06	271	896	289	37	5	3	2967
Division Teaching Hospital	3	1.49	N/A	648	172	203	73	16	21,395

RRMA: Rural, remote, metropolitan areas classification

ARIA: Accessibility / remoteness index

FTE: Full Time Equivalent

The educational process was similar to that of the teaching hospital, being based on supervised learning from patients, clinical teaching, tutorials and case demonstrations. In common with their peers at other geographical sites within the School of Medicine, the program was based on the adult learning model, requiring participating students to exercise a significant degree of self reliance and initiative. Although not essential, being a graduate rather than undergraduate program combined with an extensive use of the PBL mode of learning would have assisted this process.

A notable difference was that only 25% of the clinical material was seen at the hospital interface. Each student participated in the care of a large variety of patients, experiencing in any given week problems covering all five core clinical rotations. They were also able to follow their patients over time, creating a longitudinal experience. However, for administrative reasons, the students operated within the five-term structure, with separate 8-week blocks for each of the five core rotations. Hence, the weekly PBL tutorials, activities and assessment tasks were aligned with the designated term, an essential process to keep the curriculum and assessment process uniform across all geographical locations within the School of Medicine.

Although the GP principal in each of the practices was the primary teacher / mentor, the student's learning environment was enhanced by interactions with other locally based doctors, visiting specialists and allied health professionals. PBL tutorials were conducted by teleconference between the pilot study students and the designated tutor (either one of the GP Principals or a teacher from the Division's teaching hospital). A separate teleconference between the three students, but without supervision by the academic, teaching or administrative staff was conducted weekly to encourage peer-to-peer discussion and support. Each student also rotated to the Division's main teaching hospital or a second teaching hospital of the Division for 3–5 days per month to participate in either scheduled teaching activities with other students or one to one teaching with the hospital based specialists. This arrangement was flexible dependent on the needs of the students, in keeping with the philosophy of self-directed learning.

Each student was provided with high-speed Internet access. This gave them the same access as students elsewhere to the Internet based resources of the university, where the PBL and other learning resources are located. Each student was issued with a hand held computer and associated software, providing access to information within the consultation process and for diarizing their learning experience. This personal learning tool, akin to a learning journal was for personal self learning and development, rather than program evaluation. At no time did academic staff have access to this information. This learning tool was not provided at other geographical locations.

### The summative assessment process

In order to ensure the comparability of the pilot study students' marks with their rural and metropolitan counterparts, the assessment process was uniform across the school regardless of geographical location. Although the pilot study students were in essence in a longitudinal rather than term based program, their assessment followed the term process. In-course assessment was completed on a nominal term basis, in the sequence rural, general practice, mental health, surgery and medicine. In contrast, end of course assessment could be attempted in any order chosen by the student, as long as they completed the process on the same day as their peers elsewhere and completed exactly the same assessment. All students chose to sit the surgery assessment at the end of term 4, the medicine assessment at the end of term 5, but varied in their timing of the other 3 assessments.

The summative assessment process was essentially similar across the third year curriculum, combining a series of in-course assessments with a final written test in each of the 5 terms. Learning and assessment programs included knowledge, skills and attributes in four broad areas titled 'Domains': (1) basic and clinical sciences, (2) interpersonal and clinical skills, clinical reasoning and practice, (3) population and preventive health, and (4) ethics, personal and professional development. Hence over the course of the year, each student was extensively and repeatedly assessed in a variety of formats and settings, looking at a broad range of learning issues beyond merely acquisition of knowledge.

### Planning and implementation

The entire program was funded under the Rural Clinical School initiative of the Australian Government Department of Health and Ageing [19] and managed by the School of Medicine, University of Queensland. The preceptors were financially supported for teaching through both the Rural Clinical School initiative and a separate Australian Government program supporting teaching by General Practitioners.

The major concern during planning was to find appropriate preceptors as this program was heavily dependent on the active and dedicated participation of the preceptor in an intensive, one to one teaching and mentoring relationship. However, in the planning stages, supervisors who were essentially self-selected came forward and after the academic credentialing processes were appointed. Each supervisor was a dedicated rural doctor with greater than ten year's continuous service in that particular location. They all had extensive procedural experience although they utilized these skills to varying degrees. Each had been a supervisor of General practice trainees over many years, had supervised medical students in the past and were known to be effective teachers.

Orientation and ongoing oversight of the supervisors was performed by the program's dedicated part time academic supervisor who made frequent contact with both the students and their preceptors, both by telephone and regular face to face meetings. He was supported by part time administrative support from the student affairs officer, finance officer and regional school manager.

In order to physically accommodate students in the rural preceptors' practice, structural changes were required. These were jointly funded by the University and the preceptors. Unfortunately, the building / renovation program was not completed until after the academic year began. Student accommodation was sourced from the local real estate agents and funded by the University.

### Analysis

The Statistical Package for the Social Sciences was used for analysing the results [20]. Each student was awarded a final score for each of the five core clinical rotations, the average being the third year's global score. Marks were compared across geographic locations by analysis of variance, with Tukey post-hoc analysis. Statistical significance was defined as p < 0.05.

## Results

All three students in this pilot study completed the full year.

The marks for each rotation are detailed in table 3. All three students in the study passed each term and the year with marks well above the nominal pass mark of 60, as did all their colleagues at other rural teaching hospitals.

**Table 3:** Academic performance [for all third year students]

	n	Medicine		Surgery		Mental Health		General Practice		Rural Health		Global Score	
Mean	Std Dev	Mean	Std Dev	Mean	Std Dev	Mean	Std Dev	Mean	Std Dev	Mean	Std Dev	Mean	Std Dev

Pilot study	3	70.33	2.52	76.90	2.95	75.63	5.98	81.47	2.15	78.00	1.32	76.47	2.02
Rural: hospital based	47	65.62	8.56	71.77	5.93	79.15	8.4	82.94	6.34	79.04	6.25	75.70	4.96
Metropolitan	174	68.25	9.42	73.44	5.25	76.02	9.83	81.50	7.1	79.15	8.4	75.65	5.04

Results are percentages.

The assessment process is described in detail in the text.

There were no statistically significant differences in marks in any of the between the three students in this pilot study and those students in the rural teaching hospitals or metropolitan hospitals.

Student evaluation data showed no variation by geographic location in satisfaction with the course. However, there were differences in experiences between the pilot study students and their hospital based counterparts. Discussions with the students and their supervisors suggested that while the overall range of clinical conditions seen was essentially similar to their hospital based counterparts, there was a distinct frequency bias towards community based chronic disease. Pilot study students had the opportunity to participate in continuity of care, following the progress of individual patients over a considerable period of time. They were also able to follow their patient across multi-professional boundaries, from the community to the local hospital and in some cases accompany them to the regional hospital and participate in the surgical care they received there.

On completion of the year, all 3 (100%) students remained in the same geographical region and attended the rural clinical school. In comparison, only 7 of the 22 (32%) students attending the rural clinical school in this region in year 3, continued to stay for year 4.

## Discussion

This pilot study has demonstrated that it is possible for medical students to successfully complete the five core clinical rotations in an isolated rural general / family practice environment. However, addressing a number of apparent issues would assist further development of the program.

The design of this pilot study exposed participating students to unusual levels of isolation, especially from their peers. Research into peer support has demonstrated that peer support helps to reduce student anxiety and stress and may improve students' confidence [15,21] and that students are more likely to turn to classmates for support and help when difficulties arise [22]. It is also well recognised that one of the key concerns for undergraduate students undertaking a rural placement rotation is the geographical distance from their teachers and peers, a barrier that can result in physical and social isolation as well as the loss of peer-based learning opportunities [16]. A variety of solutions to isolation were attempted in this pilot including Internet access, regular teleconferences and visits to the rural teaching hospital. The provision of Internet access has been shown to help relieve feelings of isolation during rural placement as a medical student [17], although there is a lack of robust information evaluating electronic student support groups and peer-to-peer communication [23]. Expansion of peer support using technology, eg real time multipoint video conferencing, may further decrease the sense of isolation.

Unlike traditional teaching environments where a variety of clinical teachers contribute to teaching in any given subject, in this program a single general / family practitioner was given the onerous duty of providing the vast majority of the teaching. This was performed on a one to one basis in the context of isolated and usually very busy rural practice. Solo rural doctors could find the extra work associated with teaching to be unsustainable. However, unlike a traditional 4–6 week placement, the longitudinal nature of the program can deliver benefits to the practitioner's medical practice. Over time, the supervising GP develops a clear understanding of the strengths and weaknesses of the student and is able to allocate tasks for the student to perform under supervision that while facilitating learning are also service tasks. This can temper the workload of the supervisor, similar to the balance between service and teaching commonly seen in the post registration medical education environment.

As one person provides most of the teaching in an environment with few if any other doctors, there is no real provision for backup if the primary teacher is incapacitated or absent. Although a temporary doctor may be found at short notice to provide patient services, it is unlikely this person would be equipped to seamlessly continue the teaching program. Utilizing group practices may be one solution to this dilemma, although these were generally unavailable in the pilot study region.

Although the course was offered to 226 students, there were only three volunteers, a number quite acceptable for a pilot study of this nature. However, in a long term sustainable program of this nature, larger student numbers would be required to ensure a steady supply of students to interested practices, as there is the potential to gain or lose students at different points during the clinical years due to other factors, e.g. sickness, failure, changed family circumstances, etc. There may be economies of scale to be considered, considering the substantial infrastructure support required for the program, as well as enhanced opportunities for peer to peer interaction by distance with a larger cohort.

## Conclusion

This study has demonstrated the feasibility of teaching core clinical rotations in isolated rural areas. To maintain confidentiality, participant characteristics could not be described. Nonetheless, program effectiveness may be related to the characteristics of the self-selected participants such as gender and previous experience living and working in rural areas. Further studies, therefore, should be designed so that information about participant characteristics can be collected, reported and examined. These could also address the issues of student acceptance, teacher burnout and cost structure to assess the viability of expanding this program to a wider audience.

## Competing interests

The author(s) declare that they have no competing interests.

## Authors' contributions

SM shared executive responsibility for managing the project, analysed the results, prepared and reviewed the manuscript.

LD was a clinical tutor, participated in the conception, shared executive responsibility for managing the project, prepared and reviewed the manuscript.

VY analysed the results, prepared and reviewed the manuscript.

## Pre-publication history

The pre-publication history for this paper can be accessed here:


